# Educators’ perceptions and views of problem-based learning through simulation

**DOI:** 10.4102/curationis.v44i1.2094

**Published:** 2021-03-10

**Authors:** Sidwell Matlala

**Affiliations:** 1 Department of Nursing, Faculty of Health Sciences, University of Johannesburg, Johannesburg, South Africa

**Keywords:** simulation, problem-based, integrated learning, views, perceptions

## Abstract

**Background:**

The real-world problems and ever-changing challenges currently confronting the future of nursing education and healthcare require a problem-based learning approach using simulation strategy. This is exacerbated by the increasing burden of diseases such as tuberculosis, human immunodeficiency virus and acquired immune deficiency syndrome (HIV and AIDS) and more recently the coronavirus disease 2019 (COVID-19) pandemic, as well as advancing technology and changing regulations and policies. Problem-based learning is a student-centred learning strategy, where students are presented with situations drawn from practice, which can be used to bridge the theory–practice gap.

**Objectives:**

To explore the perceptions and views of healthcare educators on how problem-based learning can be facilitated through simulation.

**Method:**

A qualitative, exploratory, descriptive and contextual research design was used. Thirteen educators from the Faculty of Health Sciences of the University of Johannesburg, with 5 years’ teaching experience, were purposively selected from the Dean’s office, the Nursing Department, emergency medical care and the departments of podiatry, somatology and radiography. The participants were selected based on their extensive knowledge of problem-based learning and the use of simulation. Data were collected through in-depth, individual, semi-structured interviews. Thematic analysis provided six themes and 13 related sub-themes. The article focuses on the perceptions and views of educators regarding problem-based learning through simulation.

**Results:**

Problem-based learning through simulation allows students to work together in teams, which demonstrates a new modus operandi and renders a holistic approach to patient care.

**Conclusion:**

Problem-based learning through simulation should be utilised to encourage reflective knowledge exchange. Students from various departments can learn about new innovations, creativity and develop critical thinking when solving complex health-related problems.

## Introduction

The rapid changes in healthcare systems and the burdens of disease bring about new challenges for healthcare professionals. Increasing numbers of diseases such as human immunodeficiency virus and acquired immune deficiency syndrome (HIV and AIDS), tuberculosis and coronavirus disease 2019 (COVID-19) place a heavy burden on the resources in the healthcare system and nursing education institutions, affecting staffing norms and student placement in hospital wards to meet their educational objectives. The situation separates the theoretical and practical components of nursing education because students lack an understanding of how to apply the knowledge acquired from theoretical components.

For this reason, students end up having difficulties making critical analyses of nursing procedures and learning by reflection-in-action and reflection-on-action. This situation has a negative impact on teaching and learning. In order to bridge the theory–practice gap, educators from various departments suggested that something be done to augment the clinical learning environment, and some suggested problem-based learning (PBL) using simulation strategy, because 21st-century nurses should be able to function in complex technology-driven environments. Educators felt that a PBL approach would facilitate knowledge exchange and dissolve professional tribalism in the University of Johannesburg Faculty of Health Sciences. They further indicated that the interaction between PBL and simulation activities would improve reflection, problem-solving and the critical thinking of students in an integrated learning setting (Ehrenberg & Haggblom 2007:69).

Problem-based learning was first developed by McMaster University in Ontario, Canada, in 1969. The method became popular in areas such as North America, Europe and Australia in the 1970s and then spread to Asia. Problem-based learning involves the introduction of a problem in a way that is similar to its presentation in an authentic or real-life situation (Chan 2013:2299). Levine, in Ehrenberg and Haggblom (2007:69), refers to PBL as a systematic approach to resolving problems or meeting challenges that are experienced in real-life situations. The hallmark of the problem-based approach includes autonomy, independence and self-direction. Rideout et al. (2002:12) affirm that the problems provided to students for problem-solving use a range of simulation techniques and technology that shapes and directs learning while also equipping students with problem-solving skills on a practical level. The problem-based approach has been used in various healthcare departments over recent decades, but few experiences have been reported where simulation was used.

Problem-based learning using simulation assists students in acquiring teamwork skills, self-reliance, critical thinking, confidence and innovation, and the skills will thus enable nursing in South Africa to adapt to the demands of the complex future multidisciplinary working environment (Ehrenberg & Haggblom 2007:68). The method provides an authentic and comprehensive approach to healthcare education and encourages an integrated body of knowledge, where students acquaint themselves with problem-solving skills while using real-world content. Educators do not provide direct teaching but take on the role of behind-the-scenes facilitators. The approach is student-centred, meaning that students take control of their learning (Shadday 1999:369). Today’s students are faced with dynamic, evolving and complex situations that require the provision of continuous and comprehensive patient care. The participation of educators, preceptors and students in these comprehensive, coordinated and complex care situations has encouraged universities to resort to the use of simulation strategies, as this approach allows students to gain a broader understanding of their shared roles and responsibilities (Masters, Baker & Jodon 2013:173).

‘Simulation’ refers to a learning activity that provides students with the opportunity to gain valuable exposure to patient situations and practice skills in a safe environment (Masters et al. 2013:173). Simulation is a technique, not a technology, to replace or amplify real experiences with guided experiences, often immersive in nature, that evoke or replicate substantial aspects of the real world in a fully interactive fashion (Gaba 2007:126).

Simulation is essentially a strategy to facilitate learning and teaching in South Africa, where nursing education institutions are competing for clinical placement of students, especially with the current situation, where hospitals are accommodating fewer students because of the COVID-19 pandemic. As a result of this pandemic, clinical teaching and learning have been inhibited by the limitations imposed by social distancing and infection control protocols. Along with government infection prevention and control measures, the hospitals and clinics do not have enough staff to guide the students because some of them are on sick leave or are quarantined, and some of the facilities have had to be closed. Thus, nursing education institutions are left with the dilemma of accurately placing students to meet their clinical outcomes.

Participation in high-fidelity and patient simulation experiences has been found to be an effective and efficient strategy to enhance the teaching and learning processes in this period of the COVID-19 pandemic. Given that academic hospitals are often congested with students from various nursing schools, colleges and universities, a simulated PBL approach encourages students to practice skills confidently and safely. The congestion from nursing students, medical students, emergency medical care students, physiotherapy students and radiography students from various academic institutions often hinders students from meeting their educational objectives because of ward closures and the combining of wards and because nursing education institutions are only allowed to place final-year students in clinical areas. Students often compete for patients, especially in midwifery training institutions; hence, simulation is regarded as an ideal strategy to facilitate teaching and learning at this time (Masters et al. 2013:173). The University of Johannesburg Faculty of Health Sciences envisages using simulated PBL, with the primary aim of preparing all health profession students to work together to achieve the common goal of providing safe and quality care to patients.

The University of Johannesburg consists of 11 departments: nursing, radiography, sports management sciences, podiatry, somatology, biokinetics, anatomy and physiology, chiropractic, homeopathy, environmental health sciences and emergency medical care. The departments share a state-of-the-art high-fidelity simulation centre. However, the simulation centre has traditionally been used to train students in compartments, and as a result, students in clinical areas are faced with situations that are dynamic and that require departments to work outside of their silos. One of the primary goals of the Faculty of Health Sciences’ strategic imperative is to create synergy and teamwork among students from various disciplines.

The simulation was designed to provide high-fidelity technology-driven, experiential learning that facilitates multidisciplinary care using integrated learning. The centre consists of a debriefing room, two emergency or trauma rooms, four bedded adult and paediatric intensive care units, two wards and 10 multi-skill cubicles, all connected to Wi-Fi, which ensures communication across the rooms. The centre is equipped with high- and medium-fidelity patient simulators, as well as integrated audio-visual recording facilities that were built to emulate the real-world healthcare environment. Studies have shown that the use of simulated problem-based activities involving students from different healthcare professions better equips them to enter professional practice with the purpose of maximising patient care (Bandali et al. 2008:182).

Based on the vision of the university under study, the researcher undertook a research study focusing on integrated learning aimed at integrating theory and practice using a PBL strategy through simulation. According to interdisciplinarians such as Klein (2005:10), integrated learning draws its perspective from disciplines, cultures, subcultures and real-life experiences. Many integrationists regard integrated learning as a relevant approach to facilitate PBL using high-fidelity simulation because of the wide range of connection-making activities, which encompass disciplinary connections of insight and ideas. This does not replace clinical practicals, but augments and assists the students to refine their skills in a safe environment (Leonard 2012:49).

Integrated learning is a method that expands the depth and breadth of learning experiences for both learners and educators across the disciplines. It ensures that future healthcare professionals acquire the needed skills to work in complex and diverse real-life healthcare settings. The approach provides 21st-century learners with an education system that fosters both analytic and scientific thinking and with competencies that are articulated in various frameworks. Integrated learning prepares learners as global citizens who are able to perform multiple functions in complex, multidisciplinary settings. The approach produces learners who demonstrate the ability to apply acquired team-based knowledge in their working environment. It affords them the opportunity to create, reflect and synthesise thoughts. The approach enables learners and educators to make rational decisions through logical reasoning and sound clinical judgement; hence, students from various healthcare departments within the same faculty can use problem-based scenarios to solve related clinical problems using simulation (Leonard 2012:49).

Welch (2014:1) stated that integrated learning using the problem-based approach facilitates the accomplishment of a specific goal through learners working as a team, exploring and integrating ideas and insights from a variety of perspectives. This assists learners to better understand and learn the concepts of each discipline. Humes (2013:83) states that PBL affords learners the opportunity to integrate various clinical courses, disciplines and work-related experiences. Learners can perform integrative research through critical thinking, problem-solving, and inductive and deductive reasoning. The approach prepares learners for life beyond educational institutions and encourages an inquisitive attitude, as well as the capacity to connect and interpret knowledge from various perspectives. Learners can thus demonstrate humility and tolerance and learn to deal with ambiguity and value diversity.

Research has shown that 70% of learners in problem-based integrated programmes using simulation demonstrate better academic performance than learners in discipline-based programmes. The approach is considered an effective way to meet the many global clinical demands of 21st-century learners in countries such as China, Japan, Singapore, Canada, the United Kingdom, Australia and the United States of America (USA). Problem-based simulation is regarded as an ideal strategy to achieve integrated learning outcomes (Masters et al. 2013:173).

Ho et al. (2008:934) indicate that the knowledge and skills required by healthcare professionals change over time, and therefore an innovative, creative and problem-based simulation is required for healthcare students. Government agencies, academic institutions, statutory bodies, professional organisations and clinical healthcare professionals need to recognise the need for problem-based simulation activities and commit to engaging and supporting them to dissolve disciplinary boundaries and allow students to learn common skills while in the midst of outbreaks of various pandemics. This article seeks to explore and describe the perceptions and views of educators of PBL through simulation, in order to prepare students for a multidisciplinary clinical working environment.

## Methods

Permission to conduct the study was granted by the University of Johannesburg Research Ethics Committee and the Higher Degrees Committee. A qualitative, exploratory, descriptive and contextual research design was used. Thirteen educators from the Faculty of Health Sciences of the University of Johannesburg, with at least 5 years of teaching experience, were purposively selected from the dean’s office, the Nursing Department, emergency medical care, and the departments of podiatry, somatology and radiography. This study used purposive sampling that involved five male and eight female educators with more than 5 years’ experience in teaching. The participants were selected based on their use of PBL in the modules as well as simulation during the previous 5 years.

Data were collected through in-depth, individual, semi-structured interviews using an audio-recorder. Communication strategies such as active listening, probing, reflecting, clarification, paraphrasing and summarising were used. These strategies assist the researcher in obtaining in-depth information, as well as enabling participants to freely express their views, feelings and perceptions regarding the topic. The dialogue and probing questions from the interviews provided rich data regarding the educators’ perceptions and views on how PBL could be used through simulation. The questions used were derived from the results of a concept analysis of integrated learning. Participants determined the date, time and venue for their interviews, which were conducted by the researcher and took approximately 45–60 min each. The researcher ensured that the interview sessions did not interfere with the participants’ teaching activities. The consent process and purpose of the study was explained, and the educators voluntarily participated in the study.

## Data analysis

The matrix-building method of data analysis was used. The process includes data condensation, data display in matrices, and the drawing and verifying of conclusions (Miles, Huberman & Saldaña 2014:115). An independent co-coder with extensive knowledge of qualitative research assisted with the thematic coding. The researcher and independent co-coder held a consensus meeting to verify the accuracy of the identified themes and subthemes.

### Ethical considerations

Ethical approval was provided by the Faculty of Health Sciences Research Ethics Committee of the University of Johannesburg (reference number: 01-103-2015).

## Results

Six themes and 13 subthemes emerged from the data analysis process. For the purpose of this article, the following theme was used: *educators’ views and perceptions of* PBL *in simulation*.

### Educators’ views and perceptions of problem-based learning in simulation

The educators indicated that a PBL approach is the relevant strategy to use with simulation because the strategy is more integrative and team-based and involves the integration of ideas from various perspectives, compared to traditional didactic lecture methods. The educators further stated that these integrative ideas encourage teamwork and cohesion because students then perform activities together. The statement is supported by the following participant comment:

‘When students work together in teams, they demonstrate a whole new way of doing things, and render a holistic approach to patient care. The departments involved in problem-based learning should benchmark with other universities that use the same system, in order to explore new innovations that can be used to ease the congestion in classes and sessions, which allow integration of ideas and insights by teams of students and facilitators. I think for me, the best way to achieve integrated problem-based learning outcomes would be through the use of a simulation. It is an ideal strategy to collaborate with all the departments in the Faculty of Health Sciences. It creates a safe way for students to practise their required skills together before they touch a patient.’ (Participant 2, Professor in Faculty of Health Sciences, 08 January 2018)

The participant further commented:

‘A problem-based learning approach is a broad and encompassing education strategy required in class, simulation and clinical areas for problem-solving and clinical judgement. Therefore, problem-based scenarios are essential for preparation of our students for making decisions in the multidisciplinary encounters. Communication, professionalism, open-mindedness, creativity, innovation, respect and tolerance are attributes that are essential for problem-based learning. The approach ensures that learners are well-prepared and give them guidelines and rules regarding group dynamics. These types of students will be professionally matured and will definitely become all-rounders that are open to change … err … what I mean is that they will become protagonists of change or change agents in their future workplace.’

‘How it is going to be coordinated is going to be a nightmare, but if you establish a task team with assistance from the vice-chancellor, the dean and senior staff members, you will have buy-in from all the departments, and they will be able to establish a simulation task team without hesitation. This arrangement will improve interaction and inter-disciplinary communication between the departments, such as medical imaging and radiation services, nursing, emergency medical care, podiatry and other disciplines.’ (Participant 5, Clinical Facilitator, 16 January 2018)

According to educators from various disciplines, PBL is student-centred; it encourages autonomy and reflective thinking. The approach enables learners to construct their own knowledge and engage in constructive debates, thereby enhancing critical thinking. The statement is supported by the following quotation from another participant:

‘Listen to the needs of the students; allow them time to generate questions that stimulate critical reasoning and logic; give them a chance to reflect, and provide them with an opportunity to debate and support their viewpoints based on facts, giving valid reasons for their actions. As a facilitator, create an enabling environment that accommodates respect for other students’ viewpoints; these conversations enhance professional growth and maturity. Facilitators also become co-learners and create spaces where students interact and engage with each other. Facilitators can also ask students to use portfolios and reflective journals to express themselves. As facilitators of knowledge, we need to learn to listen to each other in order to improve communication; we also need mentors who will assist us to create scenarios using contemporary problems needing solutions from different perspectives. This could also be achieved through platforms such as Microsoft Teams, Blackboard, WhatsApp, chat rooms, Skype and Twitter.’ (Participant 3, Lecturer in Emergency Medical Care Department, 11 January 2018)

Other particpants concurred with the above statements, pointing out teamwork and identification of common skills as essential elements. They also suggested that PBL through simulation should start at the first- or second-year level in order for students to learn skills using real-life situations at an early stage.

‘I have done simulation scenarios with my students where I appointed an actor as a doctor, two actors as nurses and two as radiographers. They worked together, and the system worked beautifully. I provided the students with a problem that required team problem-solving and decision-making skills and asked them to write reflective notes to enable them to discuss as a team after the session. That is what made me realise that the approach should be started in first year, because that is the foundation level. The acquired knowledge and skills will build on the next level; they would understand why things are done in a certain way at an early stage; they become aware of each other’s roles, responsibilities and communication skills, for example, awareness of the dangers of radiation when the radiographers say ‘expose’. All these problem-based scenarios shape our students to fit well in the multidisciplinary context.’ (Participant 6, Lecturer in Radiography Department, 16 January 2018)

Another participant stated:

‘We need to have a simulation task team, which will arrange joint classes or core subjects with common skills, to avoid duplication. We can always do a test run and see how it fits in, and it can be incorporated into the next level of training. You need to start role-play or scenarios slowly, using a simulation activity, then identify dedicated and committed students from the same year level, preferably first or second years, but their own focus area should always be taken into consideration.’ (Participant 7, Lecturer in Nursing Department, 23 January 2018)

‘As a facilitator, encourage students to debate about real-life, contemporary situations, and see how they can reach consensus on their own. If they ask you to intervene, direct the questions back to them, to ensure that they learn to solve complex problems on their own, based on the problem provided. This affords students an opportunity to listen carefully to each other before they respond, and they learn to tackle complex problems from different angles. The professional dominance during simulation should be given attention, as it normally hinders cooperation and teamwork. The simulated scenario assists students from different disciplines to debate and share their experiences together. They learn presentation skills that prepare them for clinical rounds. I think with this system the departments will produce thinking healthcare professionals who are fit for clinical practice and fit for purpose. I think … mmm … faculty should see how they can bring scenario-based, problem-based simulation learning and research to the forefront before students are exposed to patients in hospitals.’ (Participant 7, Lecturer in Nursing Department, 23 January 2018)

Most participants suggested that educators should create an environment in which experts are included in simulated PBL activities to give guidance because they have been there before and have vast experience and exposure. They also cited consulting, benchmarking and networking with local and global universities, such as the universities of Stellenbosch and Free State, and countries such as the USA and Australia, where simulation strategy is actively used to enhance learning.

The above statement was supported by the following quotation from one educator:

‘We must encourage our students to network with other students globally; we are currently sending our students to the Appalachian State University (ASU), and they also attended Universitas 21 (U21 summer school). Our university hosted a U21 Summer School in 2017, but this is not sufficient. These integrated exchange programmes enable our students and educators to learn from their colleagues across countries by sharing and exchanging research knowledge and essential information. Our students are also encouraged to maintain academic relationships with overseas students and share their acquired insights and ideas with other students in their meetings and forums. To encourage communication, give them group work with scenarios, and assist them with questions that will stimulate dialogue, debate and argument. Allow them time and space to co-construct their knowledge, until they come up with team-based solutions to the problems.’ (Participant 4, Lecturer in Somatology Department, 12 January 2018)

Simulated problem-based scenarios in integrated learning enhance open-mindedness, logical thinking and rational reasoning. These critical thinking activities instil confidence and allow students to think creatively. Problem-based learning strategy encourages teaching and learning beyond disciplinary silos. The approach produces students who invent and design new intervention strategies, policies and protocols in the multidisciplinary clinical environment.

## Discussion

This study aimed to explore and describe the perceptions and views of educators on using PBL activities with simulation, in order to augment traditional didactic teaching strategies. Kumar and Refaei (2013:68) refer to PBL as an instructional, learner-centred approach that empowers learners to conduct research, integrate theory and practice, and apply knowledge and skills to develop a viable solution to a defined problem. It is a teaching and learning approach in which teams of students learn through problem-solving and reflecting on their experiences and existing learning. Problem-based learning using scenarios has become increasingly popular in healthcare education, and the strategy encourages student-driven learning, innovation, creativity, and independent problem-solving and decision-making skills. The participating educators agreed that integrated discussions using simulation with PBL afford students and educators the opportunity to practise their skills safely and prepare professionals to be confident in solving complex problems in their future clinical encounters (Masters et al. 2013:173).

Problem-based conversations encourage open-mindedness and ensure that people do not make assumptions and jump to conclusions before consulting with the relevant structures. Therefore, the approach requires a well-prepared and groomed educator, with extensive knowledge of teamwork activities using simulation. The educators need to understand the principles of group dynamics, and they should be able to manage interactive, technology-driven scenarios and ensure effective communication to all the team members. They should be conversant with information technology communication platforms such as Blackboard, Microsoft Teams, Zoom and WhatsApp (Ndawo, Chabeli & Nolte 2017:11).

For simulation and problem-based strategy to be effective, problems should be selected from the wide variety of current healthcare issues that affect students and their teaching and learning environment. The task teams need to determine the theoretical and clinical hours at different levels of training in accordance with regulatory requirements (Shadday 1999:370). This team approach will afford learners the opportunity to be responsible for their own learning and facilitate networking with local and global learners. Simulated problem-based scenarios encourage learners from various disciplines to identify and solve problems together through online sources and applications to engage and share information with local and global universities. Online simulated PBL strategies are essential in the current COVID-19 situation, where clinical placement of students in various healthcare facilities is becoming a challenge.

Virtual engagement may also provide students with an opportunity for knowledge exchange, which is an academic platform where learners and educators from different local and global universities share contemporary health-related issues on a rotational basis. Stakeholders and experts from various institutions have an opportunity to engage on common curricular frameworks and modules through conversations and dialogue. Using problem-based conversations encourages them to see healthcare through different lenses (Westberry et al. 2015:113).

A problem-based learning approach integrated with virtual simulation activities is used as a foundation or basis for information that cuts across disciplinary boundaries, enabling learners to engage in knowledge integration when solving complex problems locally and globally. This approach encourages reflective practice, in which learners in groups engage in self- and peer-assessment, promote change and adapt to such change in a team-based environment, which motivates them to practise as autonomous healthcare professionals. The approach encourages learners to co-construct knowledge and affords them an opportunity to make meaning from simulated scenarios as a team. The technology-driven curriculum in a virtual, simulated learning environment encourages learners to drive discussion, interaction and dialogue. They are able to question ambiguity, take initiatives to identify their learning needs and use creative, innovative strategies and relevant resources drawn from the scenarios to meet their collaborative learning needs and outcomes (Moran et al. 2015:7).

When developing a PBL strategy using simulation, the allocation of learners must be comprehensive, reflect the needs of all the students in various departments and meet the broad needs of the community that the students serve. The allocation of students should ensure that the problem-solving skills are used in different contexts and that they prepare 21st-century learners to become responsible, technologically savvy global citizens and critical thinkers (Iwasiw & Goldenberg 2015:265).

Bradshaw and Lowenstein (2011:330) asserted that knowledge and understanding of information in various disciplines encourages reflective knowledge exchange and develops a scholarly disposition of inquiry or thinking, which is required in the global village of scholars. Knowledge exchange in various disciplines exposes learners to international platforms such as conferences, webinars and research forums, in which learners and educators from various disciplines locally and globally share knowledge and experiences. Simulated scenarios also assist students and educators to achieve a deeper conceptual understanding of the integrated learning approach.

## Limitations of the study

The research design was contextual in nature, and with only one institution used for data collection, the findings cannot be generalised. Further studies on the views and perceptions of educators regarding PBL using simulation need to be explored further using quantitative studies.

## Conclusions

Simulated problem-based scenarios are relevant in the current COVID-19 pandemic situation; the strategy is appropriate because learners become involved in their own learning process. In the current situation, the healthcare departments of higher education institutions should incorporate technology to facilitate learning; this is consistent with the future needs of 21st-century learners and educators, who require virtual knowledge to engage with the world. Technology allows students to expand their horizons and ensures that they dissolve disciplinary silos and demonstrate the ability to apply their knowledge beyond classrooms (Billings & Halstead 2012:266).
